# Growth Patterns in the Irish Pyridoxine Nonresponsive Homocystinuria Population and the Influence of Metabolic Control and Protein Intake

**DOI:** 10.1155/2017/8570469

**Published:** 2017-11-15

**Authors:** Orla Purcell, Aoife Coughlan, Tim Grant, Jenny McNulty, Anne Clark, Deirdre Deverell, Philip Mayne, Joanne Hughes, Ahmad Monavari, Ina Knerr, Ellen Crushell

**Affiliations:** ^1^National Centre for Inherited Metabolic Disorders, Temple Street Children's University Hospital, Dublin, Ireland; ^2^Department of Research, Temple Street Children's University Hospital, Dublin, Ireland; ^3^Department of Laboratory Medicine, Temple Street Children's University Hospital, Dublin, Ireland

## Abstract

A low methionine diet is the mainstay of treatment for pyridoxine nonresponsive homocystinuria (HCU). There are various guidelines for recommended protein intakes for HCU and clinical practice varies. Poor growth has been associated with low cystine levels. This retrospective review of 48 Irish pyridoxine nonresponsive HCU patients assessed weight, height, body mass index (BMI), protein intake, and metabolic control up to 18 years at nine set time points. Patients diagnosed through newborn screening (NBS) were compared to late diagnosed (LD) patients. At 18 years the LD group (*n* = 12, mean age at diagnosis 5.09 years) were heavier (estimated effect +4.97 Kg, *P* = 0.0058) and taller (estimated effect +7.97 cm *P* = 0.0204) than the NBS group (*n* = 36). There was no difference in growth rate between the groups after 10 years of age. The HCU population were heavier and taller than the general population by one standard deviation with no difference in BMI. There was no association between intermittently low cystine levels and height. Three protein intake guidelines were compared; there was no difference in adult height between those who met the lowest of the guidelines (Genetic Metabolic Dietitians International) and those with a higher protein intake.

## 1. Introduction

Classical homocystinuria (HCU) (OMIM: 236200) is a recessively inherited disorder of methionine metabolism caused by inactivating mutations in the gene encoding the enzyme cystathionine *β*-synthase (CBS) (EC 4.2.1.22), leading to deficiency of the enzyme activity. CBS is necessary in the catalysis of methionine to cysteine; this pathway requires pyridoxine (vitamin B6), vitamin B12, and folic acid as cofactors. Deficiency of CBS results in the accumulation of homocysteine and methionine along with a lack of cysteine and cystine, the oxidised dimer form. There are two types of HCU: a “milder” form which responds to pyridoxine (pyridoxine-responsive HCU) and a more severe pyridoxine nonresponsive form. The incidence of HCU varies in different populations; due to a high prevalence of the G307S mutation in the* CBS *gene the incidence of pyridoxine nonresponsive HCU in Ireland is approximately 1 in 65,000 [1–4]. Newborn screening (NBS) for HCU was added to the Irish National Newborn Bloodspot screening programme in 1971.

The natural history of HCU was described by Mudd and colleagues in 1985 [5]. They reported that untreated pyridoxine nonresponsive HCU resulted in various ocular, vascular, skeletal, and central nervous system complications. They also described patients as being tall and lean [5]. Yap and Naughten [4] observed that maintaining lifetime plasma free homocysteine (fHcy) ≤ 11 *μ*mol/L (which approximates a total homocysteine (tHcy) of 100–120 mmol/L) protects against these well-recognised complications [6]. Plasma fHcy, tHcy, methionine (Met), and cystine (Cys) are monitored and tHcy and fHcy levels are lowered to within the recommended therapeutic range by dietary restriction of natural protein/methionine. Supplementation with methionine-free cystine supplemented synthetic amino acid mixtures is part of the daily treatment. Lifelong adherence to this low protein diet is recommended but is challenging and compliance issues are often encountered [7, 8].

While restriction of methionine is crucial to achieve good metabolic control, providing sufficient amounts of total protein for optimal growth and health is also needed and close dietetic monitoring is required to achieve this [1–8]. There are three widely referenced guidelines which outline a recommended total protein intake for patients with inherited disorders of protein metabolism. These guidelines include (1) the Great Ormond Street (GOS) guidelines for inborn errors of metabolism [9], (2) the Genetic Metabolic Dietitians International (GMDI) nutrition guidelines [10] which are specific to Phenylketonuria but are widely used for other protein disorders, and (3) the Ross Guidelines which are specific to HCU [11].

Case reports suggesting low Cys levels can cause poor weight gain and growth despite adequate calories have led to the hypothesis that maintaining Cys levels within the normal range is essential for growth [12, 13]. It is also recognised that elevated homocysteine concentrations are associated with low Cys levels. An increase in Cys concentration was reported to lead to a significant reduction in fHcy [14].

This retrospective study aims to examine the growth rate of Irish HCU patients from birth to 18 years, including those who were diagnosed through newborn bloodspot screening (NBS) and those who were late diagnosed (LD) before 18 years of age. We aim to examine possible associations between metabolic control (fHcy, Met, and Cys concentrations) and growth and the influence of varying protein intake recommendations on final adult height.

## 2. Patients and Methods

Ethical approval was received from the Temple Street Children's University Hospital Research and Ethics Committee, reference number 16.020. Retrospective data were collected from medical and dietetic notes and anonymised for further analysis. Parameters for metabolic control included plasma levels of fHcy, tHcy, Met, and Cys. Growth parameters included weight, height, body mass index (BMI), and midparental height. Dietetic assessments included the prescribed total protein intake including natural protein and prescribed synthetic protein intake which was calculated from dietetic notes.

### 2.1. Study Sample

All HCU patients who attend or had previously attended the National Centre for Inherited Metabolic Disorders (NCIMD) in Temple Street Children's University Hospital, Dublin, Ireland. The NCIMD is the national referral centre for children with HCU in Ireland. Patients who had pyridoxine-responsive HCU (*n* = 3) and those who were diagnosed with pyridoxine nonresponsive HCU after 18 years of age (*n* = 1) were excluded.

### 2.2. Newborn Screening

Blood samples are taken between 72 and 120 hours of life. Since 2010, the cut-off for Met levels using tandem mass spectrometry has been 50 *μ*mol/L; prior to this it was 60 *μ*mol/L. Those not diagnosed through NBS are referred to as late diagnosed (LD).

### 2.3. Data Collection

Patients were categorised as those diagnosed through NBS or those who were LD. There were nine set time points for data collection: three months, six months, nine months, twelve months, two years, four years, ten years, fourteen years, and eighteen years of age. These time points were selected as the best markers of growth as they provided a continuous log or pattern of growth through infancy, childhood, and the teenage years with emphasis on the rapid stages of growth which occur during infancy. The data for the LD group were collected at these specific time points once diagnosis was made. Serial measurements of fHcy, tHcy, Met, and Cys were recorded at each of the specified time points. Visits are scheduled such that blood samples were taken 3-4 hours after their last meal, that is, usually preprandial. The prescribed total protein intake and measurements for weight, length/height, and BMI were noted at each of the specified time points also. Data on medications prescribed such as Betaine were not included in the study design as Betaine is rarely used in our paediatric cohort as dietetic compliance is generally good and Betaine is reserved for those with poor dietetic compliance, occasionally in late adolescence.

The HCU population was compared to a non-HCU population using standard deviation scores (SDS) calculated using the LMSgrowth© Excel add-in programme [15] and compared with the British 1990 and UK-WHO growth reference data [16, 17]. Parental heights were either recorded from the patient records or taken at clinic visits and used to calculate midparental height. Midparental height is the average of both parents' heights, plotted on the appropriate height centile chart at 18 years of age after adjustment for sex.

### 2.4. Analytical Methods for Blood Sampling

Amino acid analysis of tHcy, fHcy, Met, and Cys was performed using Ion Exchange Column Chromatography with ninhydrin detection using an automatic amino acid analyser (the JEOL AminoTAC analyser, JEOL Croissy-sur-Seine, France). Lithium heparin is the preferred sample type. Separation of plasma from red cells was performed within fifteen minutes of draw and an aliquot of plasma then deproteinised using 10% by volume of 35% sulphosalicylic acid. The deproteinised supernatant was used for measurement of fHcy, Cys, and Met. Neat plasma was reduced by addition of 12% dithiothreitol prior to deproteinisation for measurement of tHcy. tHcy was measured less frequently than the other markers as it was not routinely measured in our laboratory until mid-1990s.

### 2.5. Reference Ranges and Guidelines Used

Biochemical references ranges used to categorise metabolic control are described in Tables  S1 and S2 in Supplementary Material available online at https://doi.org/10.1155/2017/8570469. The total protein intake recommended by the GOS, GMDI, and Ross guidelines [9–11] is summarised in Table  S3.

### 2.6. Statistical Analysis

Statistical analyses were conducted using the IBM Statistical Package for the Social Sciences (SPSS; Version 23, 2014, IBM, Armonk, NY). Independent categorical variables were compared using Chi squared analysis and both independent sample* T*-tests were used to compare continuous variables. A linear mixed effects model was the primary analysis comparing the population diagnosed through NBS with the LD group. Age and diagnosis were fixed factors in the model with between subject measurements treated as a random effect to account for the repeated measures aspect of the study. These models are built in R version 3-2-2 (R Core Team, 2013, Vienna, Austria). A *P* value < 0.05 was accepted for significance. For establishing the impact of other covariates on the growth process, the variables are added to the linear mixed effects model and, if significant, it can be concluded that some of the variability in size is explained by the additional covariate. Fisher's Exact Test was used as appropriate.

## 3. Results

### 3.1. Demographics

Data was collected on 48 pyridoxine nonresponsive HCU patients: 24 female (50%) and 24 male (50%). Thirty-six (75%) patients were diagnosed through NBS with 12 LD patients (25%). Current mean age in the group of patients diagnosed through NBS was 23.70 years (range 5 months to 43 years). The current mean age in the LD group was 34.64 years (range 11.97–52.59 years) with a mean age at diagnosis of 5.09 years (range 1.33–11.79 years).

### 3.2. Growth within the HCU Cohort

Overall, HCU patients had a similar birth weight compared to the general population. The mean birth weight of the male NBS cohort was slightly higher at 3.71 kg (*n* = 14); the late diagnosed group was 3.37 kg (*n* = 4) compared to 3.55 Kg for the general population. There was no difference in the mean birth weight for the female cohort which was 3.33 Kg for those diagnosed through NBS (*n* = 19), 3.36 Kg for the LD group (*n* = 3), and 3.40 Kg for the general population.

**Figure 1:**
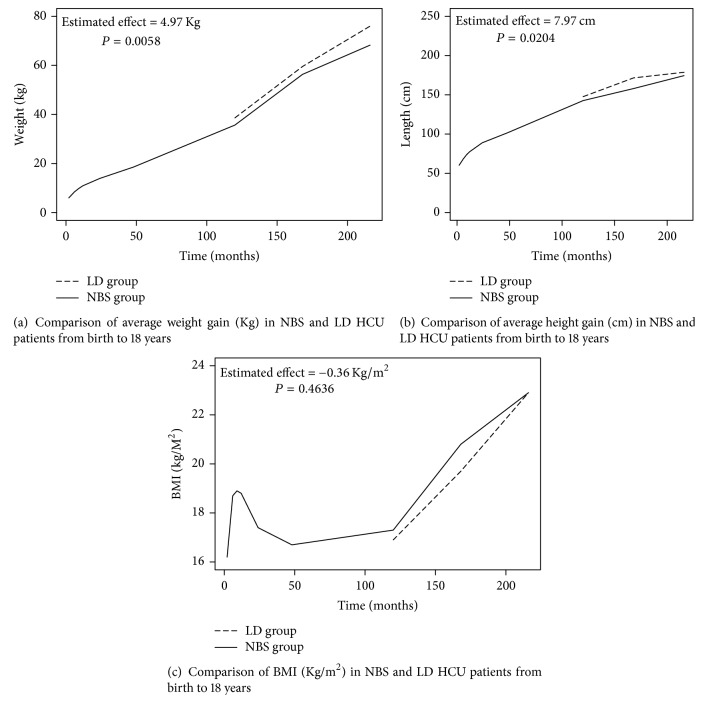


There was a significant difference in weight and height reaching at 18 years between those diagnosed on NBS and those LD. LD patients were both heavier and taller, with a weight estimated effect of +4.97 Kg (*P* = 0.0058) and height estimated effect of +7.97 cm (*P* = 0.0204). However, the growth rate between both groups beyond 10 years of age showed no significant difference. Throughout the course of the study there was an annual average increase in height of 5.95 cm for the NBS group compared to 6.85 cm for the LD group (*P* = 0.1651). Furthermore, there was an annual average increase in weight of 3.58 Kg from 10 to 18 years of age for those diagnosed through NBS compared to 4.1 Kg for the LD group (*P* = 0.621). The LD group had a slightly lower BMI (estimated effect = −0.36, *P* = 0.4636) which also increased at a slower rate from 10 years of age as illustrated in Figures 1(a)–1(c).

The difference in growth between the two groups was independent of metabolic control. When adjusted for metabolic control from ten years of age, there was no significant difference found (e.g., for Met *P* = 0.2098, for fHcy *P* = 0.2396, and for Cys *P* = 0.2432) at the nine specific age points. There was no significant association found between intermittently low Cys levels with height SDS (*P* = 0.9377).

### 3.3. Growth of the HCU Population Compared to the General Population

Those diagnosed through NBS and those LD were heavier and taller compared to the general population at nearly all ages; however, the NBS group were closer to the general population in terms of weight and height compared to the LD group; these are described in Tables S4a and S4b in the Supplementary Material. There was no significant difference in BMI between the groups suggesting a balanced increase in weight and height. This was observed in both sexes (Tables  2(a)–2(f), 4(a), and 4(b) and Figures  1(a)–1(f) in the Supplementary Material). The weight and height SDS graphs for both the male and female populations are illustrated in Figures  2(a)–2(f).

### 3.4. Mid Parental Height and Predicted Adult Height

Seventy-seven percent of LD (*n* = 10) patients and 75% of those diagnosed through NBS (*n* = 25) grew within their expected midparental height range. Two patients (15.4%) in the LD group and 6 (16.7%) of those diagnosed through NBS grew taller than their midparental height range. None of those LD and 2 (5.6%) of those diagnosed through NBS were smaller than their midparental height range at 18 years. Of those diagnosed through NBS, 7 (39%) grew as expected, 7 (39%) fell short, and 4 (24%) were taller.

### 3.5. Metabolic Control

Table 1 provides a summary of biochemical parameters taken at the defined time points in the HCU cohort. There was no significant difference in metabolic control achieved after diagnosis between the two HCU groups. The LD group had a lower number of samples recorded and the number of samples per patient was naturally skewed by their age at diagnosis.

### 3.6. Protein Requirements and Intake

Table 2 identifies the percentage of patients who achieved their full estimated protein requirement as per dietetic assessment at the specified age points according to the three guidelines which were compared. Using Fisher's Exact Test for difference, significant differences were found to exist between the numbers of patients who were achieving their estimated requirements at the various age points. However, for both the LD group and those diagnosed through NBS, regardless of whether they were meeting their estimated protein requirements or not, there was no significant difference in height at any age point (*P* = 0.5458). The highest percentage of patients met the GMDI recommended protein intake (which is lower than GOS and Ross).

## 4. Discussion

This is the largest study to date examining the growth pattern of children with HCU alongside their metabolic control. It highlights a significant difference in the way LD HCU patients grow in comparison to those diagnosed through NBS. At all ages, the LD patients were taller and heavier than those patients who had been diagnosed through NBS but this accelerated growth occurred before 10 years suggesting it may be due to poor metabolic control in infancy and early childhood prior to diagnosis. This correlates with descriptions of untreated or LD HCU patients as being taller and leaner with a “Marfanoid” appearance [1, 2, 18–20], reminiscent of the genetic condition Marfan syndrome. However, our patients are not lean; they have a similar BMI to the NBS group and the general population indicating a balanced increase in both height and weight. The future adult height of a child who is growing normally with the correct nutritional intake can be approximately predicted at 2 years of age [18, 21]. This was not observed within our NBS cohort where 39% (*n* = 7) grew within their predicted range, 22% (*n* = 4) grew taller, and 39% (*n* = 7) grew less than predicted at 2 years of age. Of note, all of our LD patients are treated aggressively with dietetic treatment and biochemical targets are the same for both NBS and LD groups. Following diagnosis, the metabolic control achieved was comparable between both groups. This highlights the importance of encouraging LD patients to initiate a protein restricted diet despite the challenges it presents. The benefits of good long term metabolic control are well established even when the diagnosis is made late in life [3, 4].

It has been hypothesised that low Cys levels lead to poor growth independent of calorie and protein intake [12, 13]. In this study we did not find any significant association between intermittently low Cys levels and height, in either the NBS or the LD groups. Gastrointestinal side effects are a frequent clinical finding following supplementation with L-cystine, a sulphur amino acid. Although Cys supplementation is not routine practice internationally, it is sometimes used for patients when Cys concentrations are persistently low despite adequate protein supply. It has been our experience that compliance with Cys supplementation is difficult due to the gastrointestinal side effects. If plasma homocysteine concentrations remain well controlled and if protein stores and growth parameters are of no concern but cystine levels are below the reference range, extra supplementation may offer little benefit.

As there are various guidelines for recommended protein intake for metabolic patients, the approaches used in different centres can vary. Protein requirements for patients with disorders of sulphur amino acid metabolism have not been individually studied so general recommendations are often used [22]. When the majority of protein sources are supplied as L-amino acids, there is rapid absorption and catabolism of the amino acid with a possible decrease of biological value. A higher total protein intake beyond age specified recommendations has therefore been recommended [23–25]. Some guidelines are condition specific; for example, Ross has published HCU specific guidelines [11], while other guidelines used are common to different amino acid-related disorders, for example, GOS and GMDI guidelines [9, 10]. These guidelines differ with GOS suggesting a much higher daily total protein intake than GMDI (Table 2). We found that significantly fewer patients achieved the GOS requirements compared to GMDI and Ross recommendations (where the suggested protein requirements are lower); however, growth was comparable irrespective of the guideline applied. Compliance with synthetic amino acid mixtures can be challenging in different age groups. Our patients had a median BMI SDS of up to 1.4 from two to eighteen years of age; therefore, using the lower requirements should be considered a reasonable option to help reduce the amount of synthetic protein prescribed and reduce unnecessary calorie intake. Synthetic protein intake in isolation may not be implicated in excessive weight gain; however, parents/patients often struggle to achieve the recommended dose and, now, knowing that the lower of the recommended guidelines does not negatively alter growth is reassuring for these patients. Further prospective studies into this area are warranted and may provide a change in practice if similar results are found.

In our NBS cohort it was more difficult to predict adult height at two years of age compared with the general population. A possible combination of different factors including environmental, genetic, hormonal, general nutrition related, and unidentified causes may be potential explanations. This warrants further investigation.

Recently published international HCU guidelines by Morris et al. [6] support the findings of this study. There is no evidence to guide cystine supplementation dose and little evidence to support the benefit of supplementation, in terms of growth. There are also no specific recommendations for synthetic or total protein intake for the HCU population. Although compliance with a protein restricted diet and methionine free L-amino acid supplement remains a lifelong challenge, good compliance and good metabolic control are essential for the prevention of complications. These guidelines also highlight the need for international collaborative studies to expand treatment options and improve our patients' health and quality of life.

## 5. Conclusions

In conclusion, the LD HCU cohort was significantly heavier and taller than the NBS cohort due to accelerated early growth but their rate of growth after 10 years of age was the same. No association was found between intermittently low Cys levels and poor growth. We conclude that achieving a higher total protein intake offers no added benefit in terms of adult height reached and patients may benefit from a lower dose of synthetic protein in terms of compliance and calorie intake.

## Supplementary Material

Figure S1a: Weight gain in the male HCU NBS, LD and general population.
Figure S1b: Weight gain in the female HCU NBS, LD and general population.
Figure S1c: Height gain in the male HCU NBS, LD and general population.
Figure S1d: Height gain in the female HCU NBS, LD and general population.
Figure S1e: Median BMI for the male HCU NBS, LD and general population.
Figure S1f: Median BMI for the female HCU NBS, LD and general population.
Figure S2a: Weight SDS for the male HCU NBS, LD and general population.
Figure S2b: Weight SDS for the female HCU NBS, LD and general population.
Figure S2c: Height SDS for the male HCU NBS, LD and general population.
Figure S2d: Height SDS for the female HCU NBS, LD and general population.
Figure S2e: Male BMI SDS comparisons for HCU NBS, LD and general population.
Figure S2f: Female BMI SDS comparison for the HCU NBS, LD and general population.

## Figures and Tables

**Table 1:** Metabolic control given as homocyst(e)ine, methionine, and cystine concentrations under treatment (*µ*mol/L).

	Free Homocystine		Total homocysteine		Methionine		Cystine	
	NBS	LD	NBS	LD	NBS	LD	NBS	LD
Number of samples	234	25	104	8	237	25	223	24
Mean	15.1	15.7	91.1	88.5	72.6	119.6	39	43
Median	9.5	5	79	71.5	62.5	99.2	31.5	35.3
Range	0–90	0–100	5–269	9–202	3–600	10–324	4–180	3–57
*P *value	*P *= 0.917		*P *= 0.933		*P *= 0.165		*P *= 0.660	

**Table 2:** Percentage of patients meeting the Great Ormond Street (GOS) and Ross and Genetic Metabolic Dietitians International (GMDI) protein recommendations.

Age	GOS	Ross	GMDI
3 months	70	70	85
6 months	52	56	85
9 months	54	75	88
12 months	54	73	100
2 years	88	96	100
4 years	61	96	100
10 years	50	100	100
14 years	90	100	100
18 years	96	100	100

*P* values using Fisher's Exact Test for difference			
Age	GOS to Ross	GOS to GMDI	Ross to GMDI

3 months	—	.004	.004
6 months	—	.041	.028
9 months	.003	.082	.010
12 months	.001	—	—
2 years	.120	—	—
4 years	.393	—	—

## Additional Points

*Suggestions.* (1) If homocysteine control is good despite being consistently low Cys levels further supplementation with cystine may not be necessary. (2) Consider reference to more than one guideline for protein requirements as following a lower total protein requirement guideline may improve compliance.
