# Supplementary material for: Growth Patterns in the Irish Pyridoxine Nonresponsive Homocystinuria Population and the Influence of Metabolic Control and Protein Intake
Source: J Nutr Metab. 2017 Nov 15;2017:8570469. doi: 10.1155/2017/8570469 (PMC5705888; doi:10.1155/2017/8570469)
Supplement: Supplementary file 1 — Figure S1a: Weight gain in the male HCU NBS, LD and general population. Figure S1b: Weight gain in the female HCU NBS, LD and general population. Figure S1c: Height gain in the male HCU NBS, LD and general population. Figure S1d: Height gain in the female HCU NBS, LD and general population. Figure S1e: Median BMI for the male HCU NBS, LD and general population. Figure S1f: Median BMI for the female HCU NBS, LD and general population. Figure S2a: Weight SDS for the male HCU NBS, LD and general population. Figure S2b: Weight SDS for the female HCU NBS, LD and general population. Figure S2c: Height SDS for the male HCU NBS, LD and general population. Figure S2d: Height SDS for the female HCU NBS, LD and general population. Figure S2e: Male BMI SDS comparisons for HCU NBS, LD and general population. Figure S2f: Female BMI SDS comparison for the HCU NBS, LD and general population. [file 8570469.f1.zip › Figure S2e Male BMI SDS comparison for the HCU NBS, LD and general population_JNME_2069814.docx]

**Figure S2e Male BMI SDS comparisons for HCU NBS, LD and general population**

…………… General population - - - - - - - - - - - NBS cohort ____________ LD cohort
